# Models for Oral Biology Research

**DOI:** 10.3390/biomedicines10050952

**Published:** 2022-04-20

**Authors:** Fernando Capela e Silva, Elsa Lamy, Paula Midori Castelo

**Affiliations:** 1 Department of Medical and Health Sciences, School of Health and Human Development, University of Évora, 7000-671 Évora, Portugal; 2 MED-Mediterranean Institute for Agriculture, Environment and Development & CHANGE—Global Change and Sustainability Institute, University of Évora, Ap. 94, 7006-554 Évora, Portugal; ecsl@uevora.pt; 3 Department of Pharmaceutical Sciences, Institute of Environmental, Chemical and Pharmaceutical Sciences, Universidade Federal de São Paulo (UNIFESP), Diadema 09913-030, Brazil; paula.castelo@unifesp.br

Oral biology is a scientific field that involves several disciplines, such as anatomy, cellular and molecular biology, genetics, microbiology, immunology, biochemistry, pharmacology, physiology and pathology. A deeper understanding of the various tissues, fluids, organs and functions of the oral cavity and craniofacial structures requires the development of new strategies, approaches and study models to prevent oral diseases, to better understand them or even propose new therapies. The advance in the knowledge of oral biology is largely due to the results of research carried out using different models—in vitro, in vivo and in silico, among others, all of them of importance for future translational and clinical applicability. 

In this Special Issue of Biomedicines, we have compiled 15 articles covering important fields of Oral Biology, namely Cariology, Periodontics, Oral Pathology, Pharmacology and Dental Materials, as commented below in this order. 

Dental caries is considered a preventable disease, but still prevalent worldwide even with advances in preventive and curative measures. For this reason, many antimicrobial and pharmacological agents have been developed for the prevention and control of this disease. In their study, Kim et al. [1] evaluated the antimicrobial activity of four known 1,4-naphthoquinones and newly synthesized pyrimidinone-fused 1,4-naphthoquinones against *Enterococcus faecalis*, *Enterococcus faecium*, *Staphylococcus aureus*, *Staphylococcus epidermidis*, *Streptococcus mutans*, *Streptococcus sobrinus*, *Porphyromonas gingivalis*, *Actinomyces viscosus* and *Fusobacterium nucleatum*. Strong antibacterial activity was found for the four known 1,4-naphthoquinones and KHQ 711, 712, 713, 715, 716 and 717 pyrimidinone-fused 1,4-naphthoquinones, suggesting that these synthetic compounds can be useful for the treatment of bacterial-associated oral diseases.

Periodontitis is a prevalent chronic inflammatory disease characterized by a progressive destruction of the tooth-supporting apparatus and is considered the main cause of tooth loss in adults worldwide; the condition can be exacerbated with the increased inflammation of the body, although no causal relation has been proven. In order to provide a deeper knowledge about the disease, Romano et al. [2] evaluated whether cytokine levels in gingival crevicular fluid (GCF) could discriminate patients suffering from stage III periodontitis with moderate (Grade B) and rapid (Grade C) progression rates before and 6 months after non-surgical periodontal treatment. The authors developed a model to identify patients with Grade C periodontitis based on the levels of cytokines IL-1β and IL-9 in the GCF, contributing to the early assessment of the risk of periodontitis progression. Furthermore, Romano et al. [3] developed a cross-sectional study, where the aim was to compare patients with untreated and treated periodontitis to assess the relationship between mineral elements in saliva and periodontal status. The authors found increased levels of copper (Cu), sodium (Na), iron (Fe) and manganese (Mn) in the saliva of patients with severe periodontitis compared to healthy ones, thus concluding that to discriminate between periodontal health and disease, the evaluation of the salivary concentration of mineral elements can be an interesting and useful approach. The oral cavity may also serve as a reservoir of *Helicobacter pylori*, although the factors required for its colonization are unknown. Kadota et al. [4], analyzed the relationship between the presence of *Helicobacter pylori* in the oral cavity and the periodontal status and levels of periodontopathogenic bacterial species. The authors observed that the periodontal pockets around the teeth, from which dental plaque samples were taken, were significantly deeper in *H. pylori* positive than negative individuals; in addition, *Porphyromonas gingivalis* was detected at a significantly higher frequency in *H. pylori*-positive individuals, although the interaction with these bacteria is not clear. 

Rankin et al. [5] evaluated the effects of N-acylethanolamines (NAEs), a family of endogenous biologically active lipid mediators, with palmitoylethanolamide (PEA), which have anti-inflammatory and analgesic properties, on oral mucosa biopsies from oral lichen planus (OLP) patients, a chronic inflammatory oromucosal disease. The authors concluded that there is an imbalance between prostaglandins and PEA in OLP, opening up the possibility that PEA might be a useful treatment for this oral disorder. In another study, Scholtz et al. [6] evaluated the changes in salivary proteins in oral cancer patients and participants with precancerous conditions such as leukoplakia (OLK) and oral lichen planus (OLP). The authors characterized some proteins and functions as a starting point for new biomarker studies. The different nature of OLK and LPO was demonstrated, showing malignant transformation and inflammation as the prominent biological processes related to OLK and LPO, respectively. Still, elevated levels of IL-6 could be detected in precancerous individuals compared to control ones. Within the scope of precancerous conditions, another study carried out by Ries et al. [7] investigated whether an increased expression of the programmed cell death ligand 1/programmed cell death receptor 1 (PD-L1/PD-1) exists in OLP and whether it is associated with malignant transformation, since it has been previously demonstrated the PD-1/PD-L1 overexpression in oral squamous cell carcinoma (OSCC) compared to healthy oral mucosa. The authors concluded that the increased levels of PD-1 and PD-L1 are related to malignant transformation in OLP, as increased PD-L1 levels might establish an immunosuppressive microenvironment, which could favor the malignant transformation and may represent a promising prognostic indicator. The study by Lequerica-Fernández et al. [8] evaluated the relevance of tumor-infiltrating lymphocytes (TILs) in OSCC. The analysis of stromal/tumor CD4+, CD8+ and FOXP3+ TILs was performed by immunohistochemistry and the possible relationships with the expression of PD-L1 tumoral and cancer stem cells (CSC) markers (NANOG, SOX2, OCT4, Nestin and Podoplanin) was evaluated. The results point out that TILs can act as biomarkers and potential therapeutic targets for OSCC cases.

Sperl et al. [9] hypothesized that many patients, to prevent allergic reactions, regularly use histamine receptor antagonists, such as cetirizine. However, these may have adverse effects on orthodontic treatment. In previous studies, the sterile inflammatory reaction underlying tooth movement appears to have been modulated by histamine. Using an animal model to compare tap water (control group) or cetirizine by daily oral gavage, they found that cetirizine dosages had no impact on the weight gain of the animals, neither the extent of tooth movement or cranial growth; additionally, root resorption and periodontal bone loss were not affected by cetirizine administration, concluding that histamine receptor antagonist cetirizine has no adverse effect that impairs orthodontic treatment and outcomes. Laurtano et al. [10] aimed to investigate how cyclosporine A and mycophenolate mofetil (immunosuppressive drugs) could interfere with the functions of human gingival fibroblasts, leading to gingival enlargement. RT-PCR was used to analyze the expression of 57 genes encoding gingival fibroblasts and demonstrated that gingival overgrowth can be caused by the chronic use of cyclosporine A and mycophenolate mofetil. However, given the contrasting data in the literature, further investigations are needed to clarify the process of immunosuppressive drug-induced gingival enlargement.

Considering the topic of Oral Biology, the blood–saliva barrier (BSB) consists of the epithelial cell layers of the oral mucosa and salivary glands, whose study is of importance to understand the transport of salivary biomarkers from blood to saliva. Lin et al. [11] established epithelial barrier models of the submandibular gland derived from the human cell line HTB-41 (A-253). Experiments with ferritin, a biomarker for iron storage, demonstrated the applicability of the selected model for transport studies, which is of great importance since cell line-based models of the epithelium of the submandibular salivary gland are still missing for this purpose. The aim of the study by Nam et al. [12] was to analyze changes in gene expression in dental pulp cells of deciduous teeth, derived from the use of pulp capping materials. Dental pulp cells were extracted and treated with mineral trioxide aggregate (MTA), Biodentine (BD) or TheraCal LC (TC). Bioinformatic analysis of differential gene expression in pulp cells exposed to BD or TC versus MTA revealed that exposure to the three different materials had no significant effect on pulp cell viability; however, the patterns of gene networks differed, suggesting that diverse functional gene differences may be associated with the use of these materials. It is assumed that orthodontic tooth movement affects blood flow in the surrounding tissues; in this way. Proof et al. [13] evaluated the vascularization in the area of tension of the periodontal ligament during experimental tooth movement in rats by magnetic resonance imaging (MRI). The authors also quantified osteoclasts and monocytes in the periodontal ligament, and the extent of tooth movement using paraffine histology and micro-CT analysis. The results of this study seem to confirm the hypothesis that perfusion is enhanced in tension zones of the periodontal ligament during orthodontic tooth movement.

Finally, within the topic of Dental Materials, Santos et al. [14] evaluated the biocompatibility of two new hydraulic calcium silicate-based sealers, named TotalFill BC Sealer (BC) and TotalFill BC Sealer HiFlow (FKG, La Chaux-des-Fonds, Switzerland) through subcutaneous implantation in connective tissue of rats. While BC exhibited the best biocompatibility performance of all tested sealers, HiFlow provided the greatest induction of mineralized tissues. Both TotalFill BC Sealer and TotalFill BC Sealer HiFlow were biocompatible and showed potential bioactivity when implanted in the subcutaneous tissue. Palma et al. [15] compared root-end preparation performed with two different ultrasonic tips, namely CVDentus and NSK, and compared the respective times spent on root preparation. The findings suggested significant differences regarding the quality of the root preparation walls, with the CVDentus tips showing better results. Regarding microcracking, as well as preparation time and marginal integrity, both ultrasonic tips showed similar results. On the other hand, the qualitative analysis showed the main morphological changes and wear of the NSK tips after use, which was not observed in the CVDentus. 
